# Volumetric Electromagnetic Phase-Shift Spectroscopy of Brain Edema and Hematoma

**DOI:** 10.1371/journal.pone.0063223

**Published:** 2013-05-14

**Authors:** Cesar A. Gonzalez, Jose A. Valencia, Alfredo Mora, Fernando Gonzalez, Beatriz Velasco, Martin A. Porras, Javier Salgado, Salvador M. Polo, Nidiyare Hevia-Montiel, Sergio Cordero, Boris Rubinsky

**Affiliations:** 1 Escuela Superior de Medicina, Instituto Politécnico Nacional, DF, México; 2 EMGS/EMM/FAM, Universidad del Ejército, DF, México; 3 Sección de Imagen Seccional-Unidad de Cuidados Intensivos, Hospital Central Militar, DF, México; 4 Departamento Ciencias de la Computación-IIMAS, Universidad Nacional Autónoma de México, DF, México; 5 Hospital General Balbuena, Secretaría de Salud del Distrito Federal, DF, México; 6 Department of Mechanical Engineering, University of California, Berkeley, California, United States of America; University of Toronto, Canada

## Abstract

Motivated by the need of poor and rural Mexico, where the population has limited access to advanced medical technology and services, we have developed a new paradigm for medical diagnostic based on the technology of “Volumetric Electromagnetic Phase Shift Spectroscopy” (VEPS), as an inexpensive partial substitute to medical imaging. VEPS, can detect changes in tissue properties inside the body through non-contact, multi-frequency electromagnetic measurements from the exterior of the body, and thereby provide rapid and inexpensive diagnostics in a way that is amenable for use in economically disadvantaged parts of the world. We describe the technology and report results from a limited pilot study with 46 healthy volunteers and eight patients with CT radiology confirmed brain edema and brain hematoma. Data analysis with a non-parametric statistical Mann-Whitney U test, shows that in the frequency range of from 26 MHz to 39 MHz, VEPS can distinguish non-invasively and without contact, with a statistical significance of p<0.05, between healthy subjects and those with a medical conditions in the brain. In the frequency range of between 153 MHz to 166 MHz it can distinguish with a statistical significance of p<0.05 between subjects with brain edema and those with a hematoma in the brain. A classifier build from measurements in these two frequency ranges can provide instantaneous diagnostic of the medical condition of the brain of a patient, from a single set of measurements. While this is a small-scale pilot study, it illustrates the potential of VEPS to change the paradigm of medical diagnostic of brain injury through a VEPS classifier-based technology. Obviously substantially larger-scale studies are needed to verify and expand on the findings in this small pilot study.

## Introduction

This research was motivated by the health needs of Mexico, where 40.7% of the population is lacking access to health services [1]. Medical imaging, the non-invasive acquisition of an image of the interior of the body, is one of the most important tools in the armamentarium of modern health services. While required for the correct diagnostic of diseases in about 20% to 30% of cases worldwide it is not available to over 60% of the world population [2], in particular the urban and rural poor [3]. Nevertheless, advanced medical imaging, such as magnetic resonance imaging (MRI) and computer tomography (CT), is often available in central urban facilities in most countries. Obviously, the use of the limited central medical facility imaging resources for first line diagnostic of the entire population is impractical in low resources environments. However, advances in wireless telecommunication and transportation have made the transportation of patients in need possible, even in parts of the world were large distances between the rural areas and central medical hubs exist; such as India. Therefore, if it becomes known that a condition requiring medical imaging has occurred, there is often sufficient time to transport the patient to a central facility with advanced medical imaging and save lives.

Diseases that require medical imaging are characterized by changes in the composition or structure of the diseased organ. Medical imaging produces a detailed image of these changes and gives a precise diagnostic of the medical condition. Our idea was that there is an intermediary diagnostic stage between the suspicion of a medical condition requiring medical imaging and performing the detailed medical imaging. We thought that it might be sufficient to know that characteristic changes occurred in the tissue of a person suspected of a particular disease, without a detailed image of those changes. If those changes are detected than the patients can be brought in for medical imaging. If not, there is no reason to perform medical imaging. This would reduce the pressure on the limited medical imaging facilities in parts of the world lacking sufficient health services while at the same time providing medical imaging services to those in true need, who otherwise would not get those services. We have developed the technology of “Volumetric Electromagnetic Phase-shift Spectroscopy” (VEPS) [4]–[6] to generate this intermediate diagnostic. This study describes the device, its’ use and reports the results of the first pilot clinical feasibility test of VEPS on patients with edema and hematomas in the brain.

Our design goal was to develop a device that is simple to use, robust, inexpensive, could be conveniently placed in parts of the world lacking medical infrastructure and which can determine in a simple way if changes that require medical imaging have occurred in a patient suspect of a certain medical condition, or not. To focus ideas we developed the technology for the detection of edema, hemorrhage and hematomas and ischemia [7]–[9]. Of course other applications, such as detection of tumors, infections, brain degeneration, internal bleeding and maternal hemorrhage may be also possible. Brain edema is a pathological condition, in which the amount of fluid in the tissue increases, usually as a function of time after an event has occurred [10]–[12]. The characteristic of brain edema is that it develops in a delayed fashion, over a period of hours or days, after a brain trauma, hypoperfusion/ischemia, bleeding/hematoma, neuroinfection or a related event has occurred. It is a cause of substantial mortality [8]–[12]. Internal bleeding in the brain causes the accumulation of blood in a certain region of the brain, which is a pathological condition known as a hematoma. The changes associated with edema and hematomas in the brain are complex, time dependent, but the end result is brain damage. Early detection and continuous monitoring of edema and hematoma in the brain is essential for assessment of the medical condition and treatment.

All the medical conditions listed above are characterized by changes in tissue composition and structure. The changes are related primarily to fluid in relation to cellular structures and in most of these conditions the changes vary in time. It is well established that the complex electrical impedance of tissue, when measured over a range of frequencies, can be used for tissue characterization [12]–[19]. This suggested to us that our design goals could be accomplished with a device that could measure through non-invasive electromagnetic induction from the exterior of the body the electromagnetic properties of the tissue in a volume of interest. To this end we conceived of a simple, inexpensive and robust device that can perform “volumetric electromagnetic phase-shift spectroscopy” (VEPS) in a volume of tissue of interest in the body from the exterior of the body [4]–[7]. Our hypothesis was that physiological changes in the volume of the targeted tissue would express themselves as changes in the VEPS measurements. Detecting and analyzing these changes could serve for diagnostic to indicate if medical imaging is needed or not.

The first stage of our research began with a theoretical work in which we applied the mathematical model of Griffiths et al. [20] on various bulk tissues and confirmed theoretically that edema in brain, lung and muscle tissue, can be detected with VEPS [21], [22]. The theoretical studies were confirmed by an experiment with *in vitro* pig brain [23]. A numerical study with circular and magnetron sensor coils has shown that it should be also possible to detect the rough location of brain hematomas with VEPS [24]. A simulation study on the ability of VEPS to distinguish between hypo-perfusion and bleeding in the brain was reported in [25]. An experimental design of the VEPS for the human head was tested on a spherical head simulation model in [25].

In the second stage we found from an *in vivo* experimental study in a rat model, that VEPS could detect abdominal accumulation of fluid in time, with high resolution [26]. A further *in vivo* study with a rat model has demonstrated that VEPS can detect temporal changes caused by ischemia in the brain [27]. The second stage culminated with a first clinical study with a group of healthy volunteers in whom tissue over-hydration was induced by ingesting 1.5 to 2 liters of water [28]. Magnetic Resonant Imaging (MRI) was used to characterize the VEPS data. Changes in the inductive phase shift at frequencies above 100 MHz were consistent with changes in the brain tissue hydration level observed by differences in diffusion-weighted (DW) MRI sequences and the apparent diffusion coefficient (ADC). The results confirmed that VEPS has the potential to detect pathologies associated with changes in the content of fluids in human brain tissue.

The third stage was made possible by recent advances in computer science and wireless telecommunication. We have shown in a series of three papers that VEPS data can be transmitted across long distances, even between continents, through wireless communication and analyzed from a distance [29]–[31]. This opens the door to the possibility for using large databases at a central facility for analysis of VEPS data taken at remote locations. We have also shown that volumetric bulk electromagnetic spectroscopy can be used to build classifiers for tissue diagnostics [32] with such applications as volumetric spectroscopic analysis of biopsies through wireless and a Support Vector Machine (SVM) classifier [33].

This paper reports results from the first small-scale pilot clinical study of VEPS for diagnostics of medical conditions in the brain. Here, multi-frequency VEPS measurements of the brain in 46 healthy volunteers were correlated with multi-frequency VEPS measurements in 8 patients with brain damage, whose condition was assessed by CT imaging. The results of the study show that the multi-frequency VEPS data can be used for diagnostic to detect brain damage and even distinguish patients with edema from those with hematoma. This is a pilot study whose size was constrained by the limited number of patients available for the study. While the results illustrate the potential value and use of the VEPS technology, it is obvious that further studies are needed to expand and confirm the findings in this paper in a much larger number of subjects.

## Materials and Methods

### Biophysical Considerations for Inductive Phase Shift Measurements

**Figure 1:**
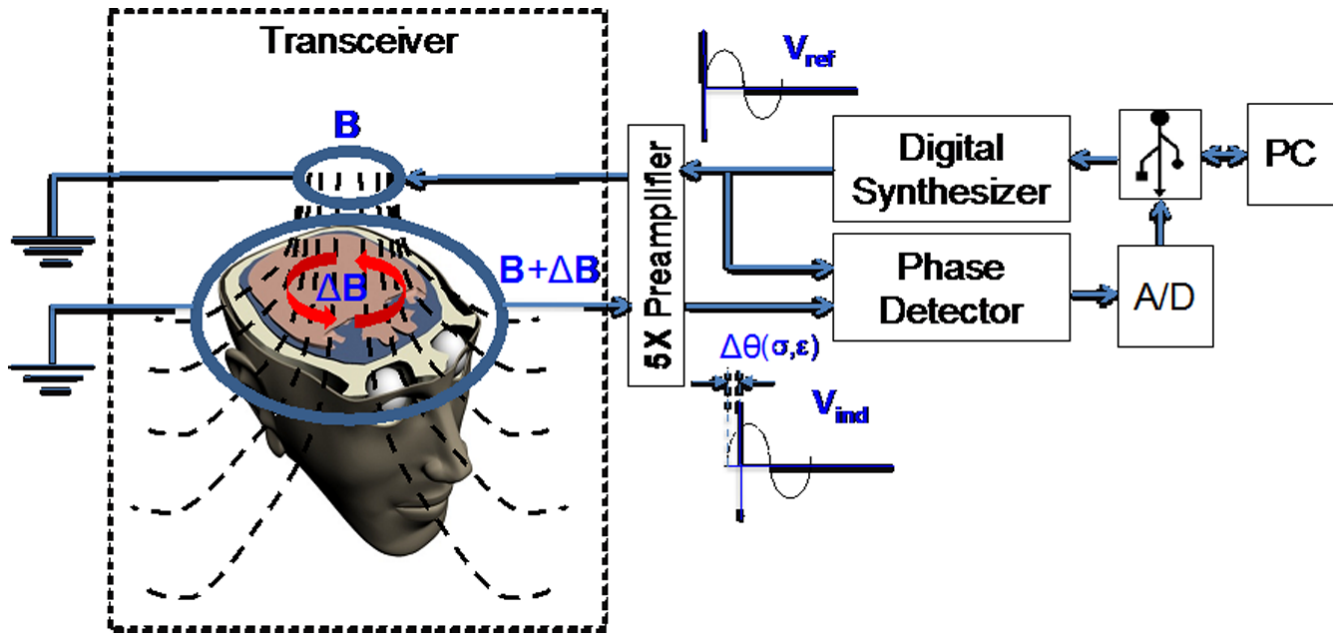
Schematic of the VEPS Head/coil configuration and a block diagram of the experimental prototype. The system consists of five modules: digital synthesizer, transceiver, phase detector, data acquisition and data processing.

A schematic of the human head/coil geometrical configuration used in this study is shown in Figure 1. The device is very simple. It consists of two coupled coils of different radii in an inductor-sensor arrangement. The coils are coaxially centered. The brain (head) is placed between the coils. An alternating current, *Ie^jwt^* is injected into the inductor coil. The current generates a primary magnetic field **B** that is detected by the sensor coil. The volume of tissue confined between the coils produces a perturbation of the primary magnetic field, (Δ**B**). The perturbation is a function of the complex impedance of the brain tissue in the volume between the coils. The perturbation is evaluated by comparing the field in the sensor coil **B**+Δ**B**, to the primary field **B**. Changes in the magnetic field represent volumetric changes in the brain composition complex impedance. A robust way to detect changes in the magnetic field is to measure the phase shift between the inductor coil and the sensor coil. Measuring the phase shift as a function of the injected current frequency produces “volumetric electromagnetic phase-shift spectroscopy” (VEPS) data. A simple way to measure the phase shift is through a “voltage relative to voltage” arrangement [27], [28]. In this arrangement the frequency dependent phase difference between the voltage in the inductor coil and the voltage in the sensor coil are used to estimate the VEPS.

### Experimental VEPS Prototype

**Figure 2:**
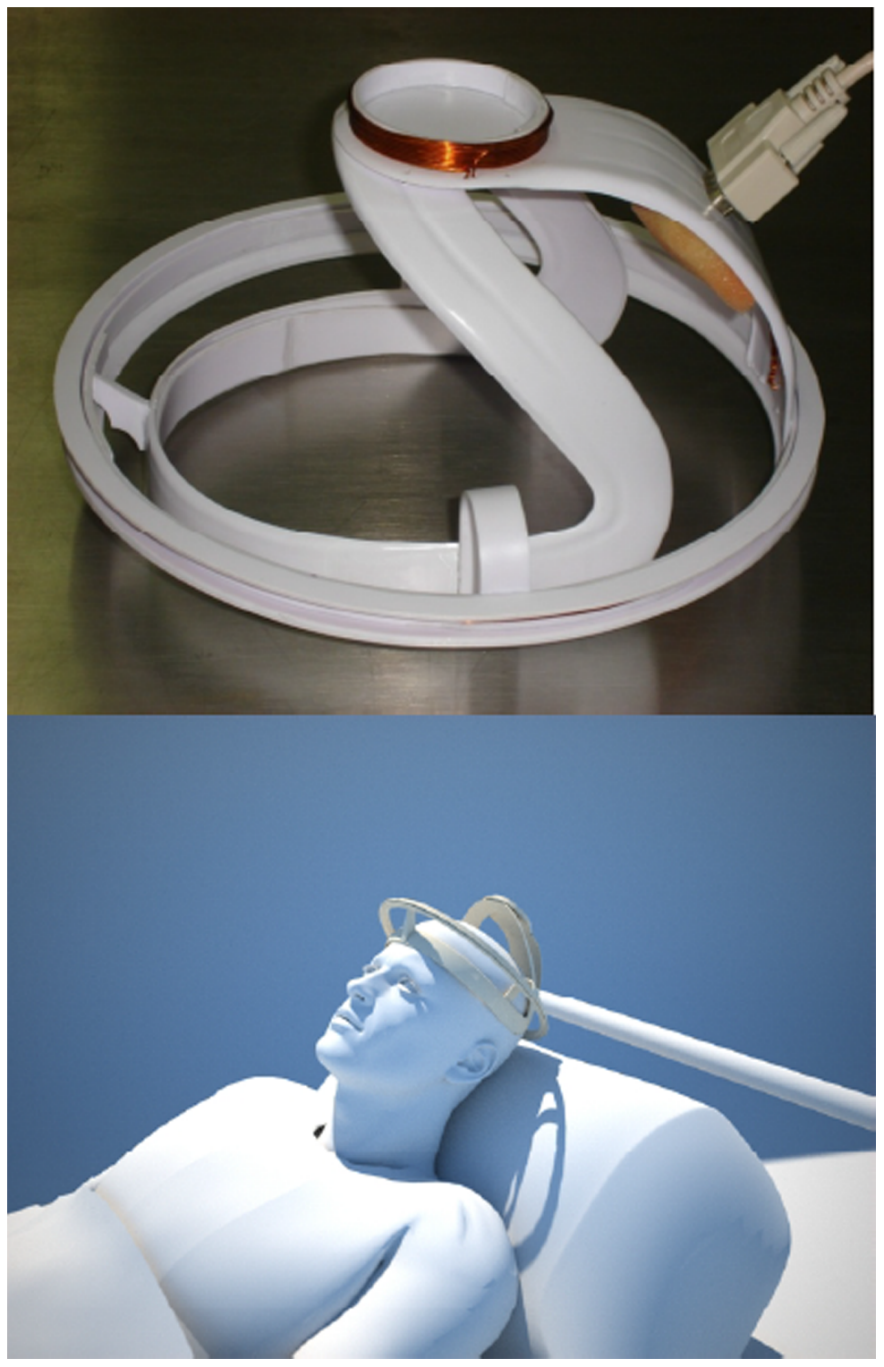
Photographs of the VEPS clinical Head/coil device and an illustration of a patient in a critical care unit wearing the device.

Following is a brief description of the VEPS data acquisition device. The system consists of five modules: digital synthesizer, transceiver, phase detector, data acquisition and data processing. The modules are shown in a block diagram in Figure 1. The digital synthesizer is a signal generator AD9958 (Analog Device Inc. Norwood, MA, USA). It supplies a sinusoidal current, Icos(ωt), of approximately 10 mA rms in the frequency range of 1–200 MHz. The current is supplied at 200 pre-programmed equally spaced frequencies, under PC control. The transceiver consists of two concentric coils with radii of R1 = 3.2 cm and R2 = 11 cm, separated by a distance of 10 cm. Both coils were built from ten turns of magnet wire AWG22 rolled on an ergonomic plastic harness specifically designed for an adult human head (Figure 2). The coil inductances, calculated from Faraday’s law, are approximately 67.4 and 796.4 µH for the inductor and sensor coils, respectively. The estimated mutual inductance coefficient is approximately M = 72.8 µH. To avoid inductive pickup the leads of the coils are twisted. A commercial device, AD8302 (Analog Devices Inc. Norwood, MA, USA) was used for phase detection. The AD8302 is a fully integrated RF IC for measuring differences in phase between two signals with a resolution of 10 mV/degree. The signals from the inductor and sensor coils are connected through a 5X preamplifier SR445 (Standford Research System Inc. Sunnyvale, CA, USA) to the digital synthesizer and phase detector module, as shown in Figure 1. The data acquisition (A/D) module uses a 10-Bit Analog-to-Digital module microcontroller 18F4550 (Microchip Technology Inc. Chandler, Arizona, USA). The VEPS data at each frequency is the average from 1024 measurements at that frequency. The sensor sample rate is 48 kSamples/sec. Photographs of the clinical VEPS inductor-sensor prototype and the way it was positioned on the head of a brain damage patient in the Critical Care Unit (CCU) are shown in Figure 2.

### Experimental Design

#### Ethics statement

**Figure 3:**
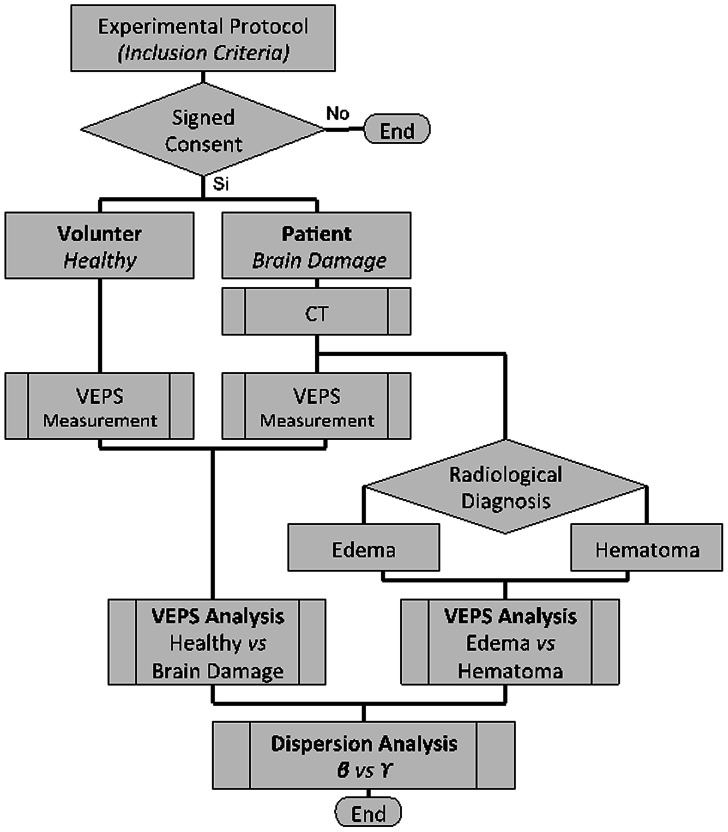
Flow diagram of the clinical study.

The study was conducted according to the principles expressed in the Declaration of Helsinki, designed by the Critical Care Unit (CCU) of the Military Hospital of the Mexican Army and was approved by both the “Research Committee” and “Bioethics Committee” of the Institution. Volunteers, patients and authorized relatives of unconscious patients were gently informed about the experimental protocol as well as its potential risks. A signed informed consent was obtained directly from volunteers and patients or relatives of the patients. The inclusion criteria were: female and males in the age range of from 18 to 70 years old without metallic prostheses or pacemakers. Figure 3 shows a flow diagram of the study. The study consists of acquiring non-invasive VEPS data from the brain of two groups of subjects: a) Healthy volunteers (46 volunteers, age 18 to 48) and b) Patients with brain damage admitted to the CCU as a result of one of the following pathologies: neuroinfection, brain vascular event or craneoencefalic trauma (8 patients, ages 27 to 70). The patients with brain damage were further classified into two typical clinical conditions with regards to the genesis of the pathology: a) *Edema -* diffuse or localized edema without hemorrhage, and b) ***Hematoma –*** **epidural, subdural, parenchymal or subarachnoid well localized hematomas.** Although hematomas are associated with edema, for simplicity we have chosen to call the condition brain-injury+hematoma, “hematoma”, because the predominant pathology of accumulation of blood. The neuroradiology department evaluated the brain pathology of the patients with computerized tomography (CT), before the VEPS study. In both, healthy volunteers and patients we measured: a) the Craneoencefalic Perimeter (CP) with a common 1 mm resolution tape and b) the multi-frequency VEPS in the range of 1 to 200 MHz at 200 pre-programmed frequencies (equally spaced) with the prototype described earlier. The multi-frequency VEPS data was normalized with respect to CP to minimize the intrinsic head volume effect on the VEPS measurements. The VEPS/CP data from healthy volunteers was compared with the data from patients with brain damage as a function of VEPS frequency and patient age. Among patients with brain damage the VEPS/CP data was compared between those diagnosed with edema and those diagnosed with “hematoma”. Because of the relatively small number of subjects, a non-parametric statistical Mann-Whitney U test was applied to the multi-frequency VEPS/CP data analysis. The statistical analysis employed the program STATISTICA V7.0 (Stat Soft. Inc.) and the significance level criteria is p<0.05.

## Results

**Figure 4:**
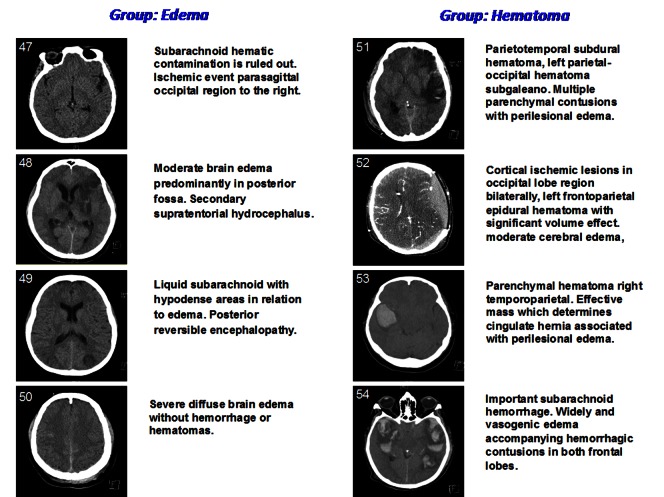
Computer tomography (CT) of the brain of the patients involved in the study, prior to the VEPS measurements. The CT’s are divide into two groups according to clinical neurology pathology valuation: *Edema* and *Hematoma*. Moderate to severe diffuse brain edema without hemorrhage or hematomas, and subdural or epidural well located haematomas regions are evident. A description of the particular pathology is given next to each CT image.

The study reported here was done with 46 healthy volunteers (ages 18 to 48) and eight patients with brain damage (ages 27 to 70). A listing of the subjects relevant personal data and their Craneoencefalic Perimeter (CP) [cm] is given in Table 1. Multi-frequency VEPS measurements were acquired with the specially build VEPS device described in the “material and methods” section and shown in Figures 1 and 2. The VEPS data from patients with brain injury was correlated with computerized tomography (CT) images of the head using the experimental protocol in the flow diagram in Figure 3. Figure 4 shows CT’s of the brain damaged patients head, divided into two groups according to their pathologies: *edema* or *hematoma*. The clinical neurological evaluation is given next to each CT image. The CT images on left (*edema*) show moderate to severe diffuse brain edema without hemorrhage or hematomas. Epidural, subdural, parenchymal or subarachnoid well-localized hematomas are seen in the images on right (*hematoma*).

**Table 1:** Table **1.** Listing of data for the healthy volunteers and brain damage patients enrolled in the study.

Group	Condition	Number	Sex	Age (yrs)	CP (cm)
**Healthy**	***Young***	1	Male	31	58
		2	Male	30	54
		3	Male	27	57
		4	Male	20	55.5
		5	Male	20	55
		6	Male	20	55.5
		7	Male	30	58
		8	Male	27	57
		9	Male	22	56
		10	Male	23	57
		11	Male	24	56
		12	Male	18	57
		13	Male	20	55.5
		14	Male	18	56
		15	Male	19	55.5
		16	Male	31	55.5
		17	Male	20	57.5
		18	Male	30	57
		19	Female	22	57
		20	Female	26	54.5
		21	Female	19	56
		22	Female	20	54.4
		23	Female	22	56
		24	Female	22	57
		25	Female	22	55
		26	Female	25	53
		27	Female	19	57.3
		28	Female	19	58
		29	Female	20	55
		30	Female	20	56
		31	Female	18	56
		32	Female	19	56
		33	Female	21	58
		34	Female	21	57
		35	Female	19	54
		36	Female	25	56
		37	Female	24	54.5
		38	Female	22	57
	***Adult***	39	Male	48	57
		40	Female	40	56
		41	Male	46	57
		42	Female	46	54
		43	Male	42	55
		44	Male	40	55
		45	Female	48	54
		46	Female	40	55
**Brain Damage**	***Edema***	47	Female	61	53
		48	Male	48	56
		49	Female	27	53.5
		50	Male	27	56
	***Hematoma***	51	Male	70	56.5
		52	Male	30	56.5
		53	Female	58	57
		54	Female	27	55

As indicated earlier, because of the relatively small number of subjects, the non-parametric statistical Mann-Whitney U test (STATISTICA V7.0 (Stat Soft. Inc) was applied to the multi-frequency VEPS/CP data analysis. The highlight of the analysis is displayed in Table 2. The non-parametric statistical Mann-Whitney U test detected statistically significant differences between the various VEPS measurements in healthy and brain-damaged subjects, with a significance level of P<0.05, in the frequency ranges from 26 MHz to 39 MHz and from 153 MHz to 166 MHz. In the frequency range from 26 MHz to 39 MHz there is a statistically significant difference between VEPS/CP in healthy volunteers and patients with brain damage. In the frequency range from 153 MHz to 166 MHz, the non-parametric statistical Mann-Whitney U test, which is designed for small number of data points, indicates statistically significant difference between the VEPS/CP measurement in patients with brain edema and those with brain hematoma.

**Table 2:** Table **2.** Statistical analysis with a Mann-Whitney U test of the VEPS/CP (degrees/cm) data for the experimental groups and subgroups in ranges of frequencies in which a statistically significant difference of P<0.05 between them, was found.

Range		Freq. (MH)	Rank Sum	Rank Sum	U	Z	p-level	Valid N	Valid N		
	* Healthy * ***vs*** * Brain damage*										
**β**		**26**	1431	54	18	4,0420	0,00005	46	8		
		**27**	1444	41	5	4,3585	0,00001	46	8		
		**28**	1398	87	51	3,2384	0,00120	46	8		
		**29**	1443	42	6	4,3342	0,00001	46	8		
		**30**	1388	43	7	4,2982	0,00002	45	8		
		**31**	1375	110	74	2,6784	0,00740	46	8		
		**32**	1386	99	63	2,9463	0,00322	46	8		
		**33**	1410	75	39	3,5306	0,00041	46	8		
		**34**	1423	62	26	3,8472	0,00012	46	8		
		**35**	1441	44	8	4,2855	0,00002	46	8		
		**36**	1449	36	0	4,4803	0,00001	46	8		
		**37**	1449	36	0	4,4803	0,00001	46	8		
		**38**	1433	52	16	4,0907	0,00004	46	8		
		**39**	1419	66	30	3,7498	0,00018	46	8		
	*Edema * ***vs*** * Hematoma*										
**γ**		**153**	11	25	1	−2.0207	0.04330	4	4		
		**154**	10	26	0	−2.3094	0.02092	4	4		
		**155**	11	25	1	−2.0207	0.04330	4	4		
		**156**	11	25	1	−2.0207	0.04330	4	4		
		**161**	10	26	0	−2.3094	0.02092	4	4		
		**162**	10	26	0	−2.3094	0.02092	4	4		
		**163**	10	26	0	−2.3094	0.02092	4	4		
		**164**	10	26	0	−2.3094	0.02092	4	4		
		**165**	10	26	0	−2.3094	0.02092	4	4		
		**166**	11	25	1	−2.0207	0.04330	4	4		

To display the results of the measurements in a concise form we calculated for each subject two parameters, β and γ. The two parameters, β and γ, are the sum of all the values of VEPS/CP [degrees/cm] in the ranges of frequencies, from 26 MHz to 39 MHz and from 153 MHz to 166 MHz, at the specific frequencies listed in Table 2, respectively.

**Figure 5:**
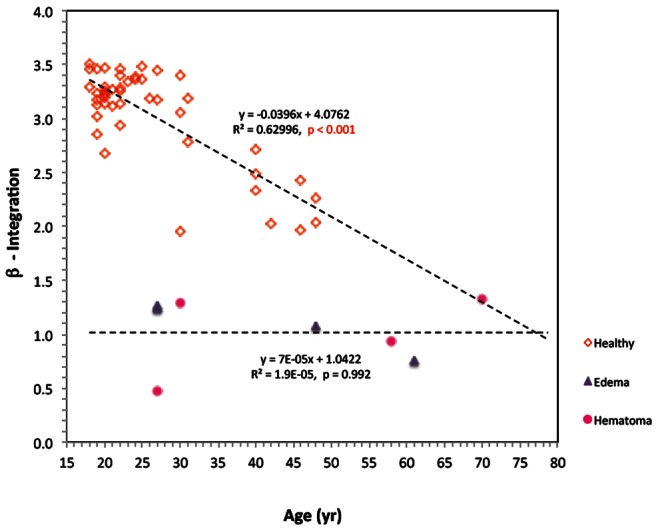
The β value for all the subjects of this study as a function of the subject age. Healthy volunteers, patients with brain condition of edema and of hematoma are marked with different symbols.

**Figure 6:**
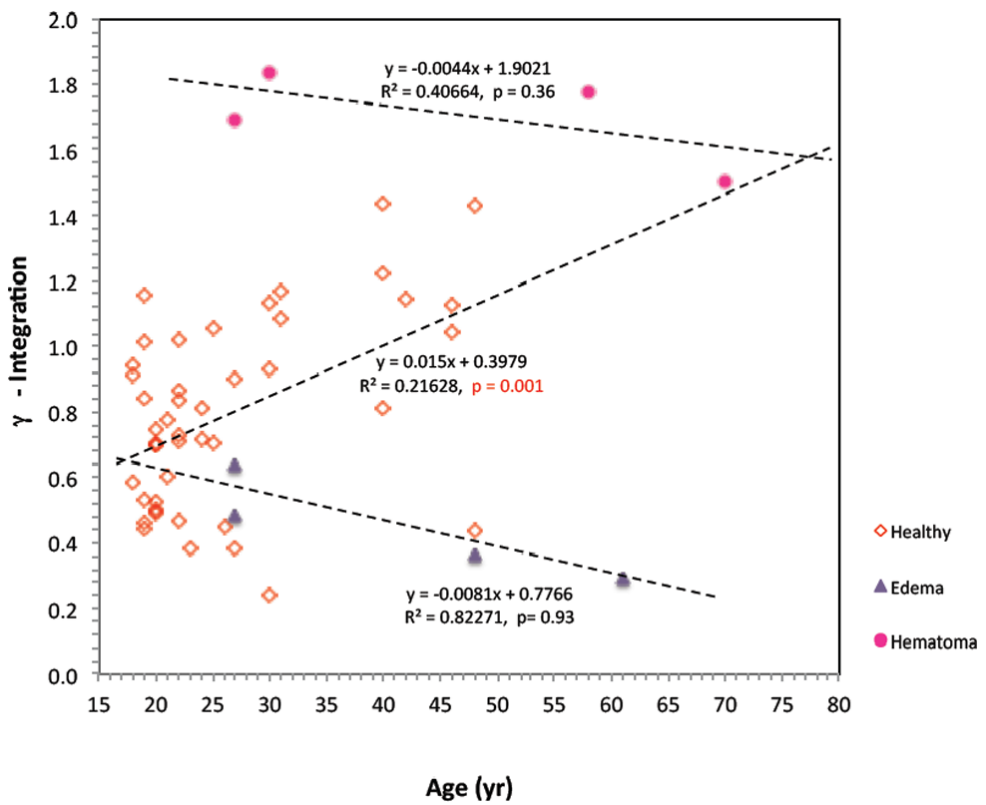
The γ value for all the subjects of this study as a function of age. Healthy volunteers, patients with brain condition of edema and of hematoma are marked with different symbols.

Figure 5 shows the β value for all the subjects of this study as a function of the subject age. It shows that in healthy individuals there is a strong correlation between the β value and age (R^2^ = 0.6299), but that in brain diseased patients there is no correlation with age (R^2^ = 1.9E-5). There is, however, a significant statistical difference between the β value of healthy volunteers and those with a brain condition as also determined from Table 2. It is interesting to note that the β value versus age curve for healthy individuals intersects with that for a pathological brain condition of edema or hematoma at an age of about 77. This suggests that the measurements of the β value alone can detect brain damage effectively in young subjects, but that it will fails for older patients. Figure 6 shows the γ value for all the subjects of this study as a function of age. It shows that in healthy individuals there is a correlation between the γ value and age (R^2^ = 0.2162), but that in brain diseased patients there is no correlation with age. Furthermore, there does not seem to be a distinction between healthy and diseased brains with age. However, as table 2 and Figure 6 indicates, there is a statistically significant difference between patients with hematoma and edema. It is interesting to notice that the correlations of the β and γ parameters with age have a different sign slope for β and γ.

**Figure 7:**
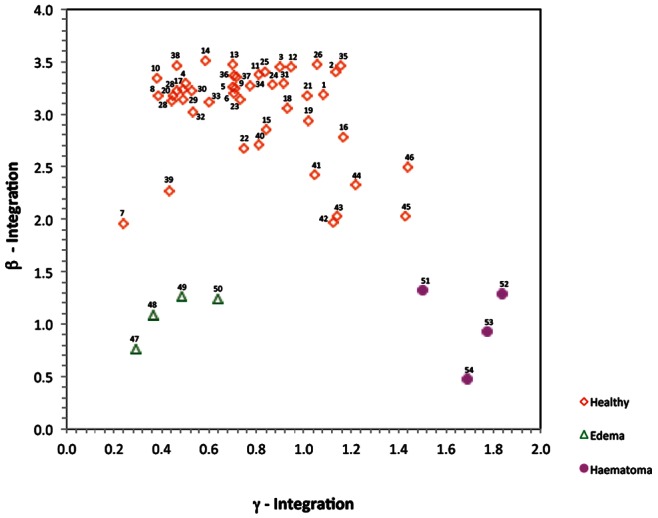
A scalar classifier plot of each experimental subject in terms of two values for that subject, β and γ. Each data point represents a subject. Healthy volunteers, patients with brain condition of edema and of hematoma are marked with different symbols.

Tables 2 and Figures 5 and 6, show that the diagnostic of the condition of the brain is a function of two VEPS parameters in the β and γ ranges of frequency. This suggested to us that a display of the data for each individual in the multi-frequency classifier modality shown in Figure 7 might have diagnostic value. Figure 7 shows the β and γ parameters for each individual in the study, represented as a data point. Each data point in the figure is identified with the subject number in Table 1. It is evident that in the representation of Figure 7, brain diseased patients stand out from the healthy volunteers and the disease modality of edema is separated from hematoma. Figure 7 bears the hallmark of a scalar classifier display. Obviously, this is a pilot study with a very small number of brain diseased patients and healthy individuals. While the results of this study are very promising much research remains to be done with a much larger number of patients.

## Discussion

The complex impedance of biological tissue displays, in the range of frequency, from DC to GHz, has three distinctive dispersions [19]. The electrical permittivity and conductivity of the three main dielectric dispersions have been labeled α, β, and γ. They occur at increasing frequencies from DC trough MHz to GHz, respectively. The α -dispersion is caused by the relaxation in the counter-ion atmosphere surrounding the charged cell membrane surface, the β -dispersion is produced by Maxwell–Wagner relaxation, an interfacial relaxation process occurring in materials containing boundaries between two different dielectrics and the γ -dispersion by the relaxation of free water within tissues [11]. Measurement of the spectral characteristics of biological tissue provides information on the structure and changes in composition of biological tissues, in particular the ratio of intracellular to extracellular fluids. The use of bioelectrical impedance measurements to detect water content and edema in the body was suggested already half a century ago [34]–[36]. Bioelectric measurements have evolved into an imaging technology known as Electrical Impedance Tomography (EIT) that uses arrays of contact electrodes to inject sub-sensory currents in the body and measure voltage to produce a map of electric impedance of tissue for use in various medical imaging applications, including detection of edema [37]–[44]. Bioelectrical measurements by electro-magnetic induction with non-contact electrical coils are considered a valuable alternative to contact electrode measurement [45]–[56]. Inductive measurement does not require galvanic coupling between the electrode and the skin or the tissue under measurement. In the particular case of brain conductivity measurement for edema detection, the skull does not represent a barrier for the magnetic field [45], [46]. This is why we have chosen non-contact electromagnetic measurements for our technology. Non-contact measurements have found applications in developing an alternative technique for electrical imaging of tissue - Magnetic Induction Tomography (MIT) and its different variants [47]–[52]. Non-contact measurements have been considered for detecting shift of water content in tissue and edema through both spectroscopy and imaging [53]–[56]. The VEPS technology that we have developed is based on the wealth of biophysical and bioengineering work from decades of previous research in the field. The novelty of our work is the concept of measuring the electromagnetic phase-shift from a *composite* volume of tissue in *a range* of relevant frequencies [4]–[6]. This leads to a very simple, inexpensive and robust device that produces spectral electromagnetic data that lend themselves to analysis with classifier technology rather than imaging. We hope that this technology can aid ameliorate the lack of health services problem faced by the many around the world, which have no access to medical imaging.

The significance of the data gathered in this experiment is best understood through table 3. VEPS measurements reflect the electromagnetic properties of a volumetric composite of various tissues. The VEPS measurement will obviously depend on the properties of each component and their relative volume in the composite. Table 3 shows that at a frequency of 25 MHz the electrical conductivity of brain tissue is about 40% of that of either human serum or blood. Obviously if in part of the volume of analysis, brain tissue is replaced by serum or blood the composite volumetric impedance in the 25 MHz frequency range will be different from that of pure brain tissue. Therefore at frequencies in the range around 25 MHz the VEPS of healthy individuals should be different from that of patients with either edema (increased human serum in the analyzed volume) or hematoma (increased human blood in the analyzed volume). This is indeed what the data in table 2 and Figure 5 show. Figure 5 brings up another observation of interest. The figure shows that the β values of healthy individuals decrease with age in a correlation with a high R^2^ value. This observation is consistent with previous animal studies, e.g. [57]–[59], which show that the electrical properties of brain tissue change with age. It is interesting to notice in Figure 5 that at the age of 77, the β values of healthy individuals approach that of brain diseased patients. This suggests that VEPS measurements made in the β range of frequencies alone may fail in diagnostic of brain conditions in elderly patients. It also indicates that the VEPS measurements may hold an as yet unrecognized insight into the human brain medical condition. As indicated several times in this paper, this is a pilot study with a limited number of subjects. However, the data is intriguing and suggests the need for further studies on the effect of age and perhaps other diseases on VEPS measurements.

**Table 3:** Electrical conductivity (S/m) at specific frequencies for brain tissue, human serum and blood.

Tissue/fluid	Frequency (MHz)		
	25	100	300
Brain (Grey Matter)	0.40	1.00	1.00
Human Serum	1.03	1.14	1.19
Blood	1.09	1.27	1.30

From^16–18^.

Table 3 shows that at frequencies of 100 MHz to 300 MHz, the electrical properties of brain tissue are substantially more comparable to those of serum and blood than at 25 MHz and different from those at 25 MHz (the dispersion phenomena). This suggests that at frequencies of 100 MHz to 300 MHz the VEPS of healthy volunteers should be similar to that of the patients with medical conditions that affect the fluid volume in the brain. This is consistent with the results plotted in in Figure 6, which show the γ values as a function of age. Figure 6 shows that while there is substantial statistical difference between the VEPS of patients and those of healthy volunteers in the β range of frequencies (Figure 5, Table 2) there is no substantial statistical difference in the γ range of frequencies (Figure 6, Table 2).

Table 3 also shows that the relative difference in electrical properties between serum and blood is larger at 300 MHz and 100 MHz, than at 25 MHz. This suggests that at these higher frequencies the VEPS should be able to discriminate between patients with edema and those with hematoma. Indeed, despite the relative small sample size Table 2 shows that there is a statistical difference between the VEPS of edema and hematoma patients in the frequency range of from 153 MHz to 166 MHz. Figure 6, confirms this. It is evident that in the β range of frequencies there is no statistical difference between patients with edema and hematoma. On the other hand, Figure 6 shows that in the γ range of frequencies the VEPS difference between patients with edema and hematoma is evident. Figure 6 also shows that the correlation between the γ value and age has a different sign slope from that of the correlation between the β value and age in Figure 5. This is an important consideration in relation to Figure 7.

Figures 5 and 6 and Table 2, show that the medical condition of the brain is a function of at least two VEPS parameters, in the frequency ranges from 26 MHz to 39 MHz and the frequency ranges from 153 MHz to 166 MHz. This suggested to us that a display of data points for each subject as a function of β and γ values of the subject might provide insight into the subject brain condition. This is a typical approach in designing classifiers [32], [33] and Figure 7 is a typical two-parameter scalar classifier display. The display in Figure 7 clearly distinguishes between the different conditions of the brain. It shows that the data points for healthy individuals, those with edema and those with hematoma are found in separate β and γ value domains. The display in Figure 7 is particularly important in relation to Figure 5. Figure 5 shows that the β value of healthy individuals decreases with age and approaches that of brain damaged individuals at age 77. This suggests that the detection of brain damage in the range of frequencies typical to β parameters may be less effective in older subjects than in younger subjects. However, Figure 7 shows that the β and γ value domains inhabited by healthy, edema and hematoma patients are distinct and there are no asymptotic changes with age as in Figures 5 and 6. This may be serendipitous and a consequence of the fact that the correlation curves in the β and γ curves with age have different sign slopes. Therefore the effect of age is cancelled in a display in terms of β and γ values and only the effect of the medical condition remains. Figure 7 illustrates the promise in building VEPS multi-frequency classifiers for non-contact diagnostic of diseases. It is very obvious that this is a pilot study with very few subjects and a larger scale study is required to validate the hypothesis suggested by this study.

It is known from clinical studies that the changes in the diseased brain are complex and occur over periods of time. From the data we anticipate that the VEPS in a patient with a medical condition in the brain will vary in time following the pattern observed here. Therefore we tentatively suggest that measuring VEPS of a patient suspected to have a medical condition in the brain could be also used as an indication for sending this patient to medical imaging at a central facility.

In summary, this is a first pilot clinical study on VEPS multi-frequency measurements in patients with edema and hematoma in the brain and in healthy volunteers. The study demonstrates that VEPS of patients with medical conditions of edema and hematoma in the brain is statistically different from that of healthy volunteers, and that it may be possible to use a simple device and a classifier display for the diagnostic of the condition of the brain.

The ability to distinguish edema in the brain from hematoma is an important finding. First it points to the sensitivity of VEPS. More important, the ability to differentiate between edema and hematoma at an early stage and even before the patient is brought to the medical imaging facility at the central hospital is of great clinical importance, as it may affect the acute treatment modality. We anticipate that when a large database of VEPS measurements will be established it should be possible to detect a medical condition directly from single VEPS measurements, through the construction of classifiers.

## Conclusion

This is a pilot clinical study in patients with a medical condition in the brain that can be detected by VEPS. The result of this first clinical study of VEPS in patients is promising. Nevertheless, this study was performed on a small number of patients and much work remains to be done to further confirm our findings. The study suggests the value of establishing a VEPS database to develop classifier methods of diagnostic. Establishing such a database is a very large effort, which is nevertheless essential for the use of this technology in the classifier modality.
